# New insights into the mechanism of substrates trafficking in Glyoxylate/Hydroxypyruvate reductases

**DOI:** 10.1038/srep20629

**Published:** 2016-02-11

**Authors:** Louise Lassalle, Sylvain Engilberge, Dominique Madern, Pierre Vauclare, Bruno Franzetti, Eric Girard

**Affiliations:** 1 Univ. Grenoble Alpes, IBS, F-38044 Grenoble, France; 2 CNRS, IBS, F-38044 Grenoble, France; 3 CEA, IBS, F-38044 Grenoble, France

## Abstract

Glyoxylate accumulation within cells is highly toxic. In humans, it is associated with hyperoxaluria type 2 (PH2) leading to renal failure. The glyoxylate content within cells is regulated by the NADPH/NADH dependent glyoxylate/hydroxypyruvate reductases (GRHPR). These are highly conserved enzymes with a dual activity as they are able to reduce glyoxylate to glycolate and to convert hydroxypyruvate into D-glycerate. Despite the determination of high-resolution X-ray structures, the substrate recognition mode of this class of enzymes remains unclear. We determined the structure at 2.0 Å resolution of a thermostable GRHPR from Archaea as a ternary complex in the presence of D-glycerate and NADPH. This shows a binding mode conserved between human and archeal enzymes. We also determined the first structure of GRHPR in presence of glyoxylate at 1.40 Å resolution. This revealed the pivotal role of Leu53 and Trp138 in substrate trafficking. These residues act as gatekeepers at the entrance of a tunnel connecting the active site to protein surface. Taken together, these results allowed us to propose a general model for GRHPR mode of action.

Glyoxylate is a small and very reactive dicarboxylic acid molecule synthesized by most eukaryotes and prokaryotes. Considered as a toxic intermediate, this compound is metabolized by an unusual enzyme assigned to the D-2-hydroxy-acid dehydrogenase superfamily, glyoxylate reductase/hydroxypyruvate reductase (GRHPR). GRHPRs are highly conserved and present in most known organisms including mammals and higher plants^1^,^2^,^3^,^4^,^5^,^6^,^7^. First, GRHPR is a dual activity enzyme. It catalyzes the reduction of glyoxylate into glycolate, which is either excreted or reconverted into glyoxylate (futile cycle) and the conversion of hydroxypyruvate into D-glycerate, a precursor supplying in the gluconeogenic pathway carbon^8^,^9^,^10^. Second, unlike most of the enzymes of the D-2-hydroxy-acid dehydrogenase superfamily, which used exclusively NADH as a co-factor, GRHPR can use either NADH or NADPH as electron donors with a certain preference for NADPH^6^,^11^.

The latter property gives an advantage to GRHPR over L-Lactate dehydrogenase (L-LDH), another D-2-hydroxy-acid dehydrogenase, which directly competes for the same two substrates. Indeed, the huge concentration of NADPH relative to NADH observed in human cytosol under normal conditions favors glycolate and D-glycerate formation by GRHPR rather than oxalate (from glyoxylate) and L-glycerate (from hydroxypyruvate) by L-LDH^11^. For this reason, the ratio of NADPH/NADH may be critical in the regulation of glyoxylate conversion because it represents a way to tune the reduction of glyoxylate by GRHPR over L-LDH^12^. When glyoxylate cannot be efficiently converted into glycolate due to a reduction in GRHPR activity, this equilibrium is disturbed, causing an accumulation of glyoxylate in cells. Consequently, this promotes the conversion of glyoxylate to oxalate by L-LDH instead of glycolate. In humans, this overproduction of oxalate in the cytosols of hepatocytes is a consequence of minor mutations in the hGRHPR gene giving rise to hyperoxaluria; a disease resulting in the deposition of calcium oxalate in the renal pelvis and kidney parenchyma^13^.

In 2006, Booth *et al*. determined the human GRHPR structure, in a ternary complex with D-glycerate and NADPH, leading to an accurate comprehension of substrate binding and active site arrangement in presence of D-glycerate^12^. Enzymatic analysis of recombinant hGRHPR shows a higher specificity constant for hydroxypyruvate than for glyoxylate, whose accumulation causes physiological disorder, suggesting that hGRHPR probably plays a key role in feeding gluconeogenesis *via* the reduction of hydroxypyruvate^11^. In contrast to the eukaryotic enzyme, GRHPR turnover for glyoxylate proceeds with higher efficiency for archaeal enzymes^1^,^6^. The GRHPR of *Thermococcus litoralis* (TliGRHPR), a hyperthermophilic archaeon, displays a higher preference for glyoxylate than hydroxypyruvate in presence of NADH, whereas no activity was detected in presence of NADPH^6^. Moreover, the GRHPR from *Pyrococcus horikoshii* (PhoGRHPR), another hyperthermophilic archaea, was described as glyoxylate reductase but with a preference for NADH cofactor. PhoGRHPR crystal structure was determined as a binary complex in presence of NADPH^1^. Previously, bacterial GRHPR from *Hyphomicrobium methylovorum* GM2 was solved in apo form^14^. Until now, no structure of GRHPR has been solved in the presence of glyoxylate, preventing an accurate description of the substrate impact on the active site as well as of the residues involved in GRHPR specificity.

For this purpose, we determined the specificity of GRHPR from *Pyrococcus furiosus* and *Pyrococcus yayanosii* (PfuGRHPR and PyaGRHPR, respectively) and correlate our results to the specificity previously determined for PhoGRHPR^1^. Unexpectedly, our results differed from those already published. This led us to fully characterize the enzymatic behavior of the three GRHPR by measuring the kinetics parameters of the four possible substrate-cofactors combinations. Although the archaeal GRHPR were previously suggested to be NADH-dependent glyoxylate reductase^1^,^6^, the present work showed unambiguously that GRHPR from *Pyrococcus* species are NADH-dependent hydroxypyruvate reductases. Finally, associated with these complete enzymatic measurements, we report here the crystal structures of both *P. furiosus* and *P. yayanosii* GRHPR enzymes. Interestingly, the structure of PfuGRHPR reveals for the first time a ternary complex in presence of glyoxylate/glycolate. This allowed the proposal of a model that explains the specificity and mode of action of this class of enzymes.

## Results

### PfuGRHPR, PhoGRHPR and PyaGRHRPR enzymes are highly thermo-activated

In order to specify the functional identity of GRHPR from *Thermococcales* species, we determined their specific activities and enzymatic parameters. Various recombinant GRHPR enzymes arising from *P. furiosus* (PfuGRHPR), *P. horikoshii* (PhoGRHPR) and *P. yayanosii* (PyaGRHPR) were produced. After purification to homogeneity, we determined their respective specific activities by using glyoxylate or hydroxypyruvate as substrates and NADH or NADPH as co-factors. The activity profile of the three enzymes, as function of temperature, was found to be similar in all substrates and co-factors combinations (see Supplementary Fig. S1 online). The three enzymes are highly thermo-activated. For enzymatic characterizations, all assays were carried out at 50 °C to slow down the reaction, especially for low substrate concentrations.

### Substrates specificities of archeal GRHPR

We first determined the initial rate of the reaction. From the initial slope of the measured curve, the reaction velocity was calculated in mol.min^−1^. The data were then fitted to a Michaelis–Menten model or to a substrate inhibition model in order to obtain the Michaelis constant (K_M_), catalytic activity (k_cat_) and substrate inhibition constant Ki (Table 1). Triplicate assays with saturating concentration of either cofactor or substrate were systematically performed.

**Figure 1:**
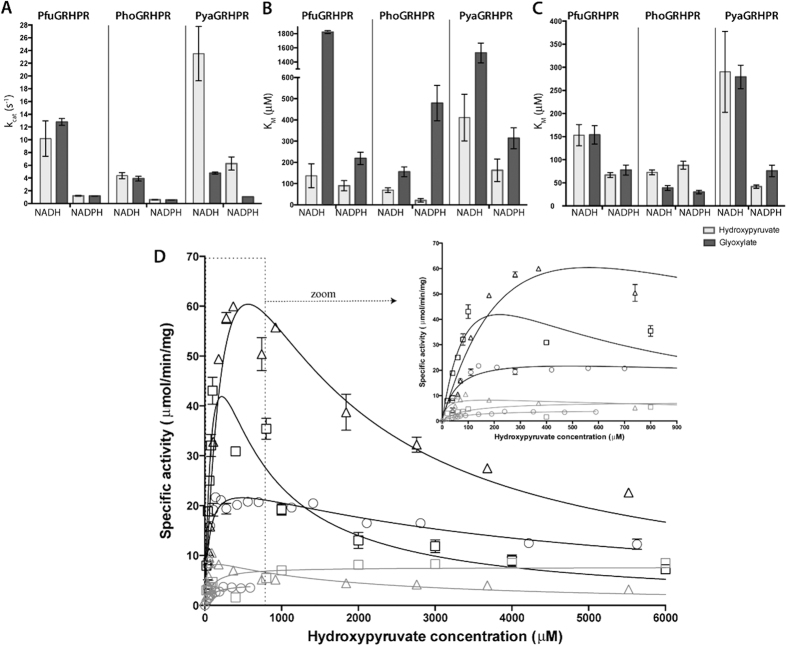
Kinetics parameters of PfuGRHPR, PhoGRHPR and PyaGRHPR. For all panels, measurements in presence of hydroxypyruvate and glyoxylate are represented in white and in black, respectively. (**A**) Catalytic activity of GRHPR enzymes for substrates. (**B**) Affinity values of GRHPR enzymes for substrates. (**C**) Affinity values of GRHPR enzymes for cofactors. (**D**) Specific activity (in mmol.min^−1^.mg^−1^) is represented as a function of hydroxypyruvate concentration (in mM) for PfuGRHPR (square), PhoGRHPR (circle) and PyaGRHPR (diamond) with NADH (black) and NADPH (gray) as cofactors. A close-up view of GRHPR activities at low hydroxypyruvate concentration (0–900 μM) is shown at right corner. Curve fit with Michaelis-Menten or substrate inhibition models are shown. Error bars are not visible when they are smaller than the font size used for the data point. Assays were performed as described under “Methods”.

GRHPR turnover for glyoxylate and hydroxypyruvate was explored (Table 1). As shown in Fig. 1A, k_cat_ values for glyoxylate or hydroxypyruvate are similar for PfuGRHPR in the presence of NADH (13.0 and 10 s^−1^ respectively) and in the presence of NADPH (1.2 s^−1^ for both substrates). A similar tendency is observed for PhoGRHPR with k_cat_ values of 4.0 and 4.5 s^−1^ in the presence of NADH for glyoxylate and hydroxypyruvate respectively, and 0.6 s^−1^ in the presence of NADPH for both substrates. For PyaGRHPR, values are higher with hydroxypyruvate than for glyoxylate in the presence of NADH (25.0 and 4.8 s^−1^, respectively) and in the presence of NADPH (6.5 and 1.1 s^−1^, respectively). For all enzymes, the highest activities were obtained with NADH, which, compared to NADPH, stimulates the enzymes activities at least 5 times (Fig. 1.A). At high (non physiological) substrate concentration, inhibition is only observed with hydroxypyruvate and NADH, except for PyaGRHPR that display also inhibition in presence of NADPH (Table 1). This behavior has already been described by (Mdlui *et al*. 2007). In order to specify the substrate specificity of GRHPR in physiological conditions, we determined their affinity parameters that, at lowest (physiological) substrate concentrations, represent the limiting factor for enzyme efficiency. As show in Fig. 1B and summarized in Table 1, the enzymes clearly exhibit higher affinity for hydroxypyruvate (20–410 μM) that for glyoxylate (160–1800 μM). PhoGRHPR exhibits the strongest affinity for hydroxypyruvate (70 μM and 20 μM with NADH and NADPH, respectively). The enzymes display a clear preference for hydroxypyruvate and can therefore be unambiguously assigned as hydroxypyruvate reductases (HPR).

### Cofactor dependence

The cofactor preference of the three enzymes was studied. Archaeal GRHPR presents higher affinity for NADPH compared to NADH (30–90 μM and 40–290 μM respectively, Fig. 1C). In the case of PhoGRHPR, K_M_ values are smaller for both cofactors in the presence of glyoxylate, whereas substrate selection doesn’t affect affinity for the cofactors in the case of both PyaGRHPR and PfuGRHPR. On one hand, K_M_ values for hydroxypyruvate are smaller with NADPH compared to NADH (Table 1), but on the other hand HPR activity of the enzymes is higher in presence of NADH. Consequently, to precisely determine the cofactor responsible for the highest enzyme efficiency, it is important to compare catalytic efficiency, k_cat_/K_M_, with respect to the substrate concentration (Table 1). As suggested by k_cat_/K_M_ ratio, NADH enhances the HPR activity. Moreover, the representation of enzymatic activity as a function of hydroxypyruvate concentration confirms that NADH enhances the HPR activity for the three considered enzymes (Fig. 1D). Based on these enzymatic measurements, it can be concluded that GRHPR from *Thermococcales* species are preferentially NADH-dependant hydroxypyruvate reductases.

### Structures determination and overall structure description

The mechanism underlying GRHPR specificity, particularly substrate discrimination between glyoxylate and hydroxypyruvate, remains elusive. To go deeper in the specificity of these enzymes, we determined the crystal structures of PfuGRHPR and PyaGRHPR at 1.4 Å and 2.0 Å resolution, respectively (Table 2). The structure of PfuGRHPR was determined by *de novo* phasing with the SIRAS method, while the structure of PyaGRHPR was determined using molecular replacement. As expected from the high sequence identity between the two enzymes (84%), PfuGRHPR and PyaGRHPR structures show close structural similarity (RMSD: 0.36 Å for monomer superposition with Secondary Structure Matching protocol as implemented in COOT^15^). For comparison, PyaGRHPR and PhoGRHPR (PDB ID code: 2DBQ)^1^ share 85% sequence identity and a RMSD of 0.46 Å. GRHPR forms a homodimer in solution^1^,^12^. For PfuGRHPR, the asymmetric unit contains a monomer, the physiological dimer being generated by the crystal symmetry operators of the I4_1_ space group. The asymmetric unit of PyaGRHPR crystal displays a full dimer.

**Figure 2:**
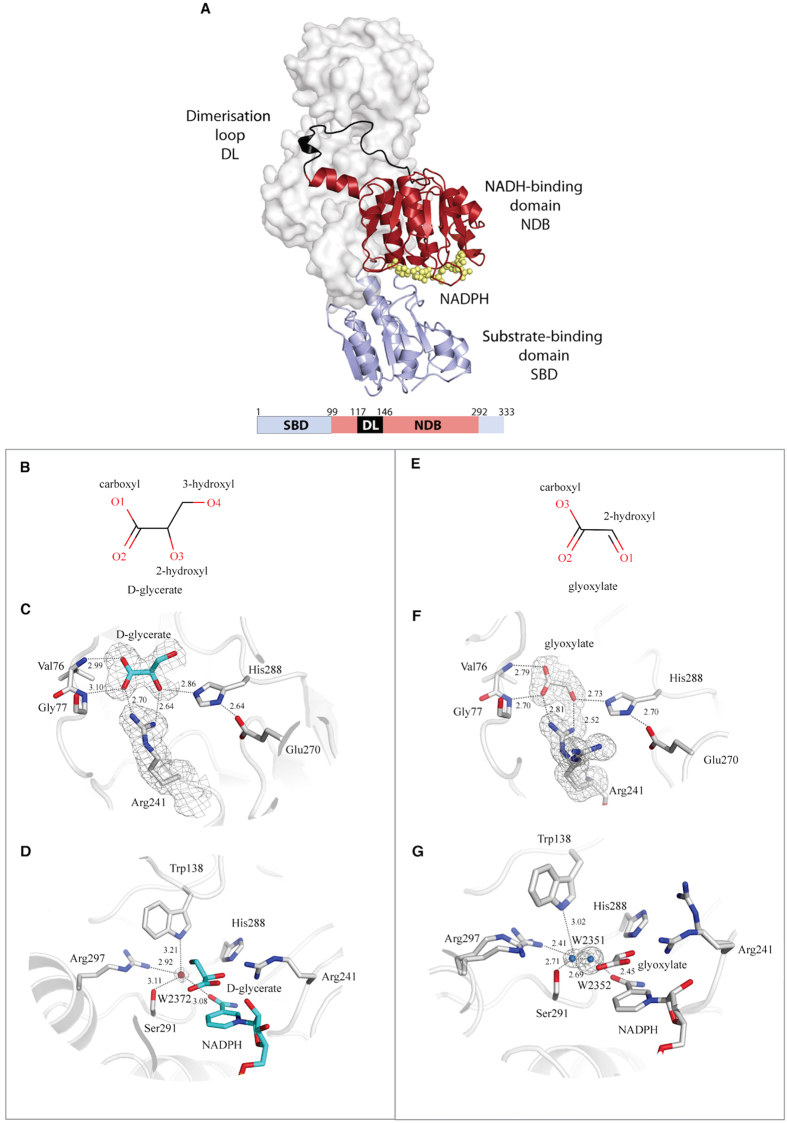
Overall structure of GRHPR. (**A**) GRHPRs are symmetrical homodimers with a large dimerization interface. One monomer is represented in cartoon while the adjacent subunit is shown as a molecular surface and white. NADPH is shown in sphere and yellow. The NADH-binding domain (residues 99–117 and 146–292), the substrate-binding domain (1–99 and 293–333) and the dimerisation loop (118–146) are represented in red, blue and black, respectively. (**B**–**D**) Views of the active sites of PyaGRHPR in presence of D-glycerate: (**B**) Skeletal formula of D-glycerate, (**C**) PyaGRHPR active site in presence of D-glycerate, (**D**) Alternative view of PyaGRHPR active site in presence of D-glycerate showing a fragment of NADPH. (**E–G**) Views of the active sites of PfuGRHPR in presence of glyoxylate: (**E**) Skeletal formula of glyoxylate, (**F**) PfuGRHPR active site residues in presence of glyoxylate, (**G**) Alternative view of PfuGRHPR active site in presence of glyoxylate showing a fragment of NADPH. D-glycerate and glyoxylate are colored in cyan and white, respectively. Superimposed sigmaA-weighted Fo - Fc OMIT map contoured at 3.0 σ depicting substrate/product, key water molecules as well as Arg241. Distance are indicated in angstrom. For PyaGRHPR the indicated distances correspond to the average of the two molecules present in the asymmetric unit (Table 3).

Each monomer of GRHPR comprises two distinctive α/β/α globular domains (Fig. 2A). These are referred to the coenzyme-binding domain (NBD) with a classical NAD(P)-binding Rossmann fold (residues 99–117 and 146–292) and the substrate-binding (or catalytic) domain (SBD), with a flavodoxin-like fold (residues 1–99 and 293–333). The active site is located in the cleft formed between the two domains. The “back-to-back” dimer involves a large interface including intermolecular contacts made exclusively between residues from the coenzyme-binding domain (Fig. 2A) that contains the dimerisation loop (residues 118–146).

A search for similar structures within the PDB using the server DALI^16^ reveals that the secondary structure of GRHPR (of PfuGRHPR as well as PyaGRHPR) shares significant similarity with phosphite dehydrogenase (PTDH) especially with PTDH from *Pseudomonas stutzeri*^17^,^18^ (PDB ID Code: 4E5N (binary complex) and 4E5K (ternary complex)). Indeed, the superposition of PstPTDH (4E5K) with PfuGRHPR or PyaGRHPR gives RMSD values of 1.32 Å and 1.29 Å with sequence identity only close to 34%. For comparison, PfuGRHPR and human GRHPR share a RSMD 1.23 Å with 40% of sequence identity with the same comparison method. The secondary structures of each domain are remarkably conserved between GRHPR and PTDH enzymes. In the present article, residues numbering refers to *Pyrococcales* GRHPR sequences.

### Cofactor binding site

The electron density maps obtained revealed that Pfu and PyaGRHPR enzymes are in ternary (enzyme + cofactor + substrate/product) form. Neither cofactors nor substrates were added during the crystallization or the cryoprotection step. Both structures include a cofactor molecule, unambiguously modeled as NADP(H), within each active site with a refined occupancy of 100%. The binding site of NADP(H) is located adjacent to the interface between the two domains and oriented as previously described for human GRHPR^12^ and PhoGRHPR^1^. Indeed, GRHPR contains a consensus sequence (Gly-X-Gly-X-X-Gly) involved in dinucleotide binding^19^. This glycine-rich loop (residues 157–162) recognizes the pyrophosphate moiety of the cofactor. Even though the binding of cofactor is similar in human and archaeal GRHPRs, a few differences exist at the vicinity of NADPH 2′-phosphate group. In the hGRHPR structure, the 2′-phosphate group is located in a pocket formed by Arg184 and Arg188. In the *Pyrococcales* structures, Arg184 is replaced by a lysine. This substitution may provide an advantage for NADH over NADPH. Moreover, in hGRHPR, arginines 184 and 188 are surrounded by two prolines (Pro183 and Pro185). In contrast, the PyaGRHPR structure possesses only one proline whereas PfuGRHPR and PhoGRHPR none. Compared to hGRHPR, this may provide more flexibility to this region that have been related to the enzyme specificity for NADH or NADPH^12^.

### Comparison of D-glycerate binding in human and archaea GRHPR

The PyaGRHPR structure contains D-glycerate, product of HPR activity as observed in the holo form of human GRHPR^12^ (Fig. 2C). The observed electron density for the hydroxypyruvate substrate didn’t allow us to distinguish unambiguously between substrate and product (see Methods). However, we can speculate that the PyaGRHPR crystal structure contains the product rather than the substrate as proposed for human GRHPR structure^12^. Indeed, purification includes a heat shock treatment at 85 °C. During this first purification step, the enzyme may catalyze the hydroxypyruvate substrate which derives from *Escherichia coli* metabolism.

Superposition of both monomers A of the PyaGRHPR structure and the hGRHPR structure (PDB 2GCG) using the program COOT^15^ gives a RMSD of 1.18 Å, despite the low sequence identity (42%). Contrary to what was observed in hGRHPR structure, both active sites in the PyaGRHPR dimer are occupied with a D-glycerate molecule. The interactions occurring in the substrate binding site of PyaGRHPR are consistent with those previously described^12^. A conserved histidine, His288, forms the acid/base catalyst and is held by Glu270 that helps to maintain the pKa of the histidine (Fig. 2C). The conserved Arg241 was proposed to orient and to hold the substrate for catalysis through two hydrogen bonds with the 2-hydroxyl and carboxyl groups of D-glycerate respectively^1^,^12^. Additional interactions for substrate orientation are provided through carboxylate oxygen atoms forming charged hydrogen bonds with the main chain amines of Val76 and Gly77 (Fig. 2C). Moreover, Leu53, through its CD2 methyl group, forms a van der Vaals contact with D-glycerate. The interaction distances in the active site of PyaGRHPR and hGRHPR are similar (Table 3). The only difference between the PyaGRHPR and hGRHPR active sites occurs in the vicinity of Ser291 and Trp138 from the adjacent monomer. Indeed, in the hGRHPR structure, the hydroxymethyl group of D-glycerate interacts with Ser291 and Trp138 *via* a conserved water molecule (W3169 in PDB 2GCG) (Fig. 2D). Although this water molecule is present in the PyaGRHPR structure (W2372), it does not mediate the interaction of D-glycerate with Ser291 (Fig. 2D). In fact, the hydroxymethyl group of D-glycerate interacts directly with Trp138 (Fig. 2D). Finally, the interaction between D-glycerate and Ser291 is abolished in PyaGRHPR structure.

### Description of GRHPR active site with glyoxylate

For the first time, the determination of the PfuGRHPR structure reveals a ternary complex in the presence of glyoxylate. Indeed, the electron density showed unambiguously a smaller substrate compared to D-glycerate and was assigned to a glyoxylate molecule with full occupancy (Fig. 2F) (see Methods). As for the PyaGRHPR crystal structure, the glyoxylate derives from overexpression in *E. coli*. However, it is difficult to provide a tentative explanation for its presence, since no substrate inhibition is observed with it (Table 1).

Glyoxylate is located and oriented as D-glycerate in PyaGRHPR structure (Fig. 2C). As expected, the majority of the interactions between glyoxylate and PfuGRHPR enzyme involves the same residues as those implicated in D-glycerate binding and previously described in PyaGRHPR and hGRHPR structures (His288, Arg241, Gly77 and Val76). As shown in Table 3, the interaction distances, between glyoxylate and residues that hold it (Arg241, Gly77 and Val76), are globally smaller compared to the distances in presence of D-glycerate.

The main difference between glyoxylate and D-glycerate is the additional 3-hydroxymethyl group in D-glycerate. Previously, Booth *et al*. describes a network of interactions between the Arg297 guanidinium group, the Ser291 hydroxyl group, the Trp138 indole ring nitrogen and the NADPH nicotinamide amide oxygen *via* a conserved water molecule^12^. This network interacts with the substrate molecule *via* the 3-hydroxymethyl group and has been suggested to be involved in GRHPR specificity^12^. With the presence of the same substrate in the PyaGRHPR structure (Fig. 2D), this network, involving Arg297, Ser291 and Trp138, is conserved and superposes strictly with the one observed in the hGRHPR active site, including the conserved water molecule. On contrary, this network is disturbed in presence of glyoxylate molecule. Indeed, absence of hydroxymethyl group induces a modification of the interactions within the network. As shown in Fig. 2G, two water molecules, separated by 2.69 Å, replace the single water molecule observed with D-glycerate. Within the network, the interactions are split between the two waters, Trp138, Arg297 and Ser291 interact with W2351 whereas NADPH and Ser291 interact with W2352. Additionally, a double conformation for Arg297 in the presence of glyoxylate is observed (Fig. 2G). The alternative conformation doesn’t interrupt the interactions because the nitrogen atoms are globally located at the same place as Arg297 in presence of D-glycerate, but this alternative conformation could change the substrate environment. The disruption of this network related to the physicochemical properties of glyoxylate could be associated with the poor affinity of PfuGRHPR and PyaGRHPR enzymes for it (Fig. 1B).

### Existence of a tunnel connecting the protein surface to the active site

**Figure 3:**
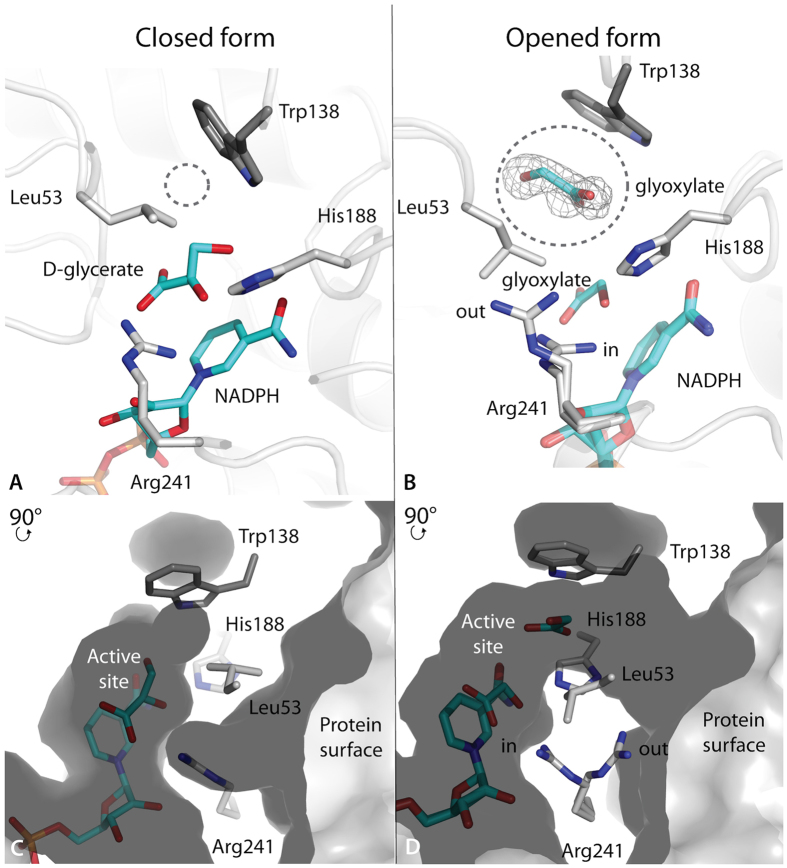
Active site accessibility in PyaGRHPR and PfuGRHPR structures. Top panel, details of the catalytic residues showing the cofactor as well as substrates, D-glycerate (**A**) and glyoxylate (**B**). The tunnel is indicated with a gray circle. In Panel (**B**), the second glyoxylate molecule is represented within the tunnel with the cage representing simulated-annealing sigmaA-weighted Fo - Fc OMIT map contoured at 3 sigma. Bottom panel, cross section of the surface representation showing the tunnel in closed form (**C**) and in opened form (**D**). Substrate and cofactor molecules are represented with sticks in cyan. Panels (**A**,**C**) are from PyaGRHPR structure with D-glycerate and panels (**B**,**D**) are from PfuGRHPR structure with glyoxylate.

Comparison between GRHPR structures with D-glycerate or glyoxylate bound highlights the differences in the conformations of residues of the active site. Unexpectedly, the catalytic Arg241, which is supposed to orient the substrate molecule^12^, is present in two conformations in the presence of glyoxylate (Fig. 3). One is basically the same as for D-glycerate, pointing toward the subtrate (“in” conformation, Fig. 3A). On the contrary, the other conformation points out at the surface of the protein with the lateral chain oriented out of the active site (“out” conformation, Fig. 3B). Even more, Leu53 adopts a different conformation that keeps it away from Trp138 and end up close to Arg241 (Fig. 3B). Additionally, the presence of glyoxylate led to the observation of a shift out the active site by 1.20 Å of Trp138 (Fig. 3B), through a global movement of the protein main chain. Consequently, the distance between Trp138 and Leu53 is larger in the presence of glyoxylate, leaving extra space (4.75 Å and 6.95 Å with D-glycerate and with glyoxylate, respectively). Indeed, additional electron density was observed close to Trp138 that cannot be associated to mother liquor components or water molecules. A second glyoxylate molecule could be perfectly assigned to the observed electron density and was successfully refined with 50% occupancy (Fig. 3B).

The impact of these alternative conformations on the spatial arrangement of the catalytic pocket was analyzed. This led us to identify a tunnel connecting the catalytic pocket to the exterior of the protein (Fig. 3D). This tunnel is form by Met52, Leu53, Ser54, Tyr74, Ala75, Leu100, Trp138 (from the adjacent monomer), Glu270, Met300, Arg297 and Arg241 and closed by the NADPH molecule in the back. The aperture toward the exterior of the protein point out in the opposite side of the hinge between the two domains, close to the dimerisation loop that carry on Trp138. This tunnel wasn’t described before because it is formed in the closed form of PyaGRHPR and hGRHPR structures due to alternative conformations of Trp138 and Leu53 (Fig. 3C).

## Discussion

In the present work, the enzymatic results indicate unambiguously that GRHPRs from *Pyrococcus* species are hydroxypyruvate reductases. Indeed, measurements of HPR and GR activities clearly show a higher catalytic efficiency for hydroxypyruvate compared to glyoxylate for all the studied enzymes. The Michaelis constant (K_M_) of PyaGRHPR for hydroxypyruvate is always smaller, when compared to the K_M_ for glyoxylate, whatever the cofactor, with similar values as determined for human GRHPR^11^,^12^. This may explain why the structure of PyaGRHPR, detailed in the present article, is a ternary complex (D-glycerate-NADPH-enzyme) providing a second example of a GRHPR structure together with human GRHPR^12^ (PDB ID code: 2GCG). The comparison of these two structures shows that the orientation and location of D-glycerate are identical with similar distances between substrate and catalytic residues. In the PyaGRHPR structure in the presence of D-glycerate, the network Trp138, Ser291 and Arg297 superposes strictly with the one identified in the human structure^12^. Booth *et al*. have proposed that this network regulates substrate binding especially for hydroxypyruvate molecule^12^. A conserved water molecule located at the center of this network appears to interact with the hydroxymethyl moiety and the residues involved in the network. Our data show therefore that both human and archaeal GRHPR exhibit a common binding mode of D-glycerate.

For the first time, the structure of a GRHPR has been determined in presence of glyoxylate in the active site and shed light on the process of substrate discrimination by GRHPR enzymes. No clear indication from kinetic parameters could explain the presence of glyoxylate. However, this unique structure provides new elements to discuss the specificity and the affinity of GRHPR enzymes. The comparison of archaeal structures of GRHPR with D-glycerate (PyaGRHPR) or with glyoxylate (PfuGRHPR) shows that the glyoxylate molecule is globally located and oriented as D-glycerate. Nonetheless, the absence of a hydroxymethyl moiety in the glyoxylate molecule (compared to D-glycerate) reduces the number of interaction between glyoxylate and the protein and induces shorter interactions distances. This may induce a mis-positioning of the substrate leading to the observed reduced glyoxylate reductase activity for archaeal GRHPRs.

The network, encompassing Arg297, Ser291 and Trp138, has been proposed to be involved in the control of substrate selection in GRHPR enzymes, interacting with D-glycerate *via* the hydroxymethyl group^12^. In PfuGRHPR structure, this interaction is obviously abolished with glyoxylate. Despite a different substrate, phosphite dehydrogenase (PTDH) possesses a high structural similarity with GRHPR and the active-site residues are strictly conserved^17^,^18^. In PTDH, systematic mutations of the three residues composing the network (Arg297, Ser291 and Trp138) show a clear effect on substrate affinity^20^. Similarly, it can be argued that, in GRHPR, these residues are involved in substrate selection through regulation of the substrate affinity. This could be related with the poor glyoxylate affinity for the enzyme in archeal GRHPR compared to hydroxypyruvate one.

Previously, Booth *et al*. pointed out the presence of a water molecule making multiple contacts with the D-glycerate, the NADPH and the network^12^. In PTDH, this water molecule is conserved and potentially acts as a nucleophile^20^. The strong impact of the Arg297 mutation on PTDH activity has led to the proposal of an activation role of this residue for the conserved water molecule. In presence of glyoxylate, this conserved water molecule isn’t detectable. Two water molecules replace it and Arg297 adopts a double conformation. The rearrangement of water molecules in GRHPR active site could allow small adjustments in substrate binding mode. However, the precise role of these water molecules in GRHPR activity remains to be clearly established.

We analyzed the structural features that control the cofactor dependency in GRHPR enzymes. In human GRHPR, it has been shown that cofactor nature has strong effects on the kinetic parameters^11^. While NADPH tends to promote enzyme affinity to substrate (3 to 4-fold), NADH enhanced k_cat_ values 2-fold^11^. Mdluli *et al*. have concluded that hGRHPR has a higher catalytic efficiency (k_cat_/K_M_) for both substrates with NADPH relative to NADH^11^,^12^. The favored cofactor for the enzyme from the archaeal organism *P. horikoshii* has been shown to be NADH^1^. By taking into account kinetic parameters obtained as a function of substrate concentration, our study confirms that archaeal GRHPRs, including PhoGRHPR, are NADH dependant enzymes. Indeed, NADH enhanced k_cat_ values for the three enzymes of the Archeal species, up to 10 fold for PfuGRHPR.

Archeal and human GRHPR structures have been solved with a NADPH molecule in the active site according to the high affinity of all GRHPR for NADPH^1^,^11^. Furthermore, presence of NADPH tends to enhance GRHPR affinity for the substrate. As with hGRHPR, archeal GRHPR possess an arginine, Arg188, close to the negative 2′-phosphate moiety of NADPH that has been shown to be critical for nicotinamide cofactor specificity in other dehydrogenases^12^,^21^. Moreover, the substitution of Lys184 (archeal GRHPR) in Arg184 (human GRHPR) strengthens the positively charged environment around the negative 2′-phosphate moiety of NADPH and could be associated with the higher affinity of human GRHPR for NADPH compared to archeal one.

In 2010, a structure of human GRHPR in apo form, at 2.82 Å resolution, was deposited in the Protein Data Bank (PDB ID code: 2WWR). Moreover, in the structure determined by Booth *et al*.^12^, both binary (NADPH) and ternary forms are present within the asymmetric unit (PDB ID code: 2GCG). Consequently, for hGRHPR, both catalytic forms (apo and holo) are available. It gives us the possibility of comparing the angular variation between the two domains composing GRHPR and potentially relates the resulting movement with substrate and/or cofactor bindings. Indeed, we observe a closing motion of SBD domain relative to NBD domain leading to a reduced angular distance between apo and binary form. This motion induces a closing of the substrate-binding site. No extra closing is observed between the binary and ternary forms as already mentioned by Booth *et al*.^12^. In conclusion, cofactor binding initiates enzymatic reaction by forming a competent active site through the relative movement of the two GRHPR domains.

Detailed analysis of substrate interactions in the PfuGRHPR structure described in this paper shows the existence of a tunnel that controls the substrate trafficking. Indeed, this tunnel connects the active site to the protein surface. Up to now, this tunnel has never been described due to alternative conformations of residues involved in it. Indeed, in presence of D-glycerate, the entrance of the active site is obstructed as observed in hGRHPR and PyaGRHPR. In particular, this tunnel is surrounded by Leu53, Trp138 and Arg241 and is large enough to accommodate a substrate molecule as illustrated by the presence of an additional glyoxylate molecule close to Trp138, providing a view of the substrate pathway. Arg241 has been proposed as a contributor to substrate orientation^12^. The observed double conformations of Arg241 in presence of glyoxylate suggests an additional role. These conformations may provide the two extreme positions of Arg241 and a view of the pathway that would be associated to carry one substrate molecule from the protein surface to the active site. As Arg241 interacts with keto and carboxylate oxygens present in both substrates, the substrate-guiding role would be relevant for both HPR and GR activities. Additionally, the structures described in this paper show that the tunnel has opened and closed forms associated with conformational changes of Trp138 and Leu53. In the conformation observed in PyaGRHPR and hGRHPR structures (with the tunnel in the closed form), Leu53 has been proposed to be involved in GRHPR specificity by preventing pyruvate binding^12^. A new conformation of Leu53 has been modeled in PfuGRHPR structure (with the tunnel in the opened form). These observations suggest that Leu53 is not only involved in substrate selectivity but is also associated with tunnel opening/closing. Moreover, as already mentioned, Trp138 acts on GRHPR specificity by interacting with hydroxymethyl moiety of hydroxypyruvate molecule favouring HPR activity^12^. This residue, provided by the adjacent monomer, is located on the dimerisation loop. Displacement of the dimerisation loop is related to the closing/opening of the tunnel through a lateral movement as illustrated by Trp138. This suggests that Trp138 and the dimerisation loop could be involved in allosteric regulation of the GRHPR dimer.

**Figure 4:**
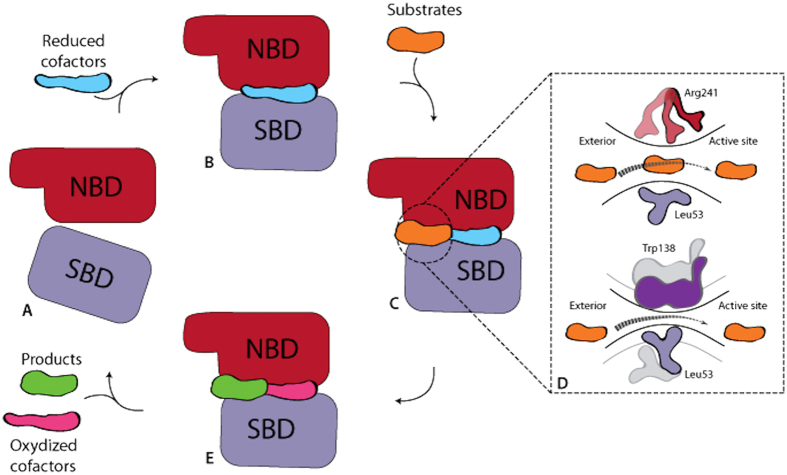
Model of catalytic process in GRHPR. (**A**) Schematic representation of GRHPR monomer with NBD domain in red and SBD domain in mauve. This corresponds to the apo form of the enzyme. (**B**) Binding of the cofactor induces a domains movement and formation of the tunnel. (**C**) Substrate binding. (**D**) Residue rearrangements associated with substrate trafficking. (**E**) Product release.

Analysis of domain movements associated with cofactor binding as well as new insights in the role of catalytic residues allows to propose a general model for the catalytic process of GRHPR as illustrated in Fig. 4. From the apo enzyme (Fig. 4, Panel A), the cofactor binding leads to the closure of active site through a relative movement of the two domains constituting the enzyme (Fig. 4, Panel B). The closure of the active site formed a tunnel connecting the protein surface to the active site. This tunnel allows the substrate to enter the active site protein (Fig. 4, Panel C). The Arg241 acts as a guide for substrate entering and substrate progression is facilitated by residues movement of Leu53 and Trp138 that regulates opening/closing of the tunnel (Fig. 4, Panel D). Substrate optimum position within the catalytic pocket is then raised thought interactions with catalytic residues (His288, Arg241, Val76 and Gly77).

In conclusion, the results presented here showed unambiguously that the GRHPR from *Pyrococcus* species are in fact NADH-dependent hydroxypyruvate reductases with a residual glyoxylate reductase activity. For the first time, the GRHPR structure was solved in presence of glyoxylate. The detailed analysis of this unique structure has highlighted the presence of a tunnel that we proposed to be involved in substrate trafficking. Based on these new insights, a model of the catalytic process of GRHPR is proposed.

## Methods

### Protein overproduction and purification

For production of recombinants PfuGRHPR, and PhoGRHPR and PyaGRHPR genes were generated from synthetic DNA fragments optimized for codon usage in *Escherichia coli* and cloned in the overexpression plasmid pET41c by GeneCust Europe. BL21 (DE3)-RIL cells containing the respective plasmid were grown with shaking at 37 °C overnight in LB medium [20 g of LB broth (Sigma) l-1 of deionized water] containing 50 μg/ml kanamycine. Ten milliliters of this subculture was added to 1 l of LB medium supplemented with kanamycine to a final concentration 50 μg/ml. After incubation with shaking at 37 °C until the A600 reached 0.6–1.0, the induction was carried out by adding isopropyl-β-D-thiogalactopyranoside to a final concentration of 0.5 mM, and shaking for a further 4 h. Cells were harvested by centrifugation for 15 min at 4500 g, and the pellet was resuspended in 50 mM Tris (pH 7.5) and 20 mM NaCl containing 0.1% Triton X-100.

After treated with Lysozyme 0.25 mg/ml, DNaseI 0.05 mg/ml RNAse 0.2 mg/ml, cOmplete Protease Inhibitor Cocktail Tablets (Roche) and MgSO_4_ (0.01 mM), the disruption of the cells was achieved by sonication on Branson sonifier 150 3 times for 30 s with intermediary pauses of 30 s on ice. The crude extract was heated at 85 °C for 30 min and then clarified by centrifugation at 17000 *g* for 45 min at 4 °C.

The supernatant was loaded on a 6 ml Resource Q column (GE Healthcare) equilibrated with 20 mM Tris-HCl, 0.05 M NaCl, pH 7.5. After washing with 3 column volumes (CV) with this buffer, proteins were eluted at 3 ml min^−1^ with a 20 CV linear salt gradient (from 0.05 to 0.25 M NaCl in 20 mM Tris-HCl pH 7.5). Fractions of 3 ml were collected and those with GRHPR activity were pooled and concentrated using an Amicon cell (Millipore) with a molecular mass cutoff of 30 kDa. The protein was loaded onto a Superose 12 column (GE Healthcare). An elution peak corresponding to an apparent molecular mass of 66 kDa was observed. According to SDS-PAGE and mass spectrometry, the corresponding fractions contained pure GRHPR. The fractions were pooled and kept at 4 °C after concentration to about 10 mg/ml.

### Enzymatic assay

Activity measurements were carried out in a UV-Vis 660 Jasco spectrophotometer (France) equipped with a Peltier-effect thermoregulated cell (20–100 °C). Activity was followed by NAD(P)H absorbance at 340 nm.

The effect of temperature on the activity was measured in the range from 50 °C to 90 °C with a prewarmed buffer containing sodium phosphate buffer 50 mM (pH 8.0). For the four possible substrate/cofactor combinations, concentrations of glyoxylate or hydroxypyruvate (0.2 mM) with NADH or NADPH (0.32 mM) were used with different protein concentrations depending on temperature: PfuGRHPR (3.1–20.0 μg/ml), PhoGRHPR (0.4–20.0 μg/ml) and PyaGRHPR(0.2–20.0 μg/ml). Each measurement was done in triplicate. Results are summarized in Supplementary Fig. S1 online (using GraphPad Prism version 6.00 for Mac, GraphPad Software, San Diego California USA, www.graphpad.com).

To determine the kinetic parameters, the initial velocity was examined by varying the concentration of one substrate while keeping the concentrations of the other substrates constant. A prewarmed buffer containing Tris-HCl 100 mM (pH 8.0) was used. Concentrations of glyoxylate (0.05–8 mM) and NADH (0.5 mM) or NADPH (0.5 mM) and hydroxypyruvate (0.05–8 mM) and NADH (0.5 mM) or NADPH (0.5 mM) were used. Enzyme concentration was determined by absorbance at 280 nm. Different range of protein concentrations were used according to the cofactor: in presence of NADH, 0.4, 1.6 and 0.4 μg/ml for PfuGRHPR, PhoGRHPR and PyaGRHPR respectively and in presence of NADPH, 1.2, 9.2 and 3.3 μg/ml for PfuGRHPR, PhoGRHPR and PyaGRHPR respectively. The data were fitted to a Michaelis–Menten equation or substrate inhibition equation to extract the values of the Michaelis constant K_M_, turnover number k_cat_ and substrate inhibition constant using GraphPad Prism version 6.00 for Mac, GraphPad Software, San Diego California USA, www.graphpad.com. Values with their standard errors are summarized in Table 1. Each measurement was done in triplicate. All enzymatic tests were carried out at 50 °C.

### Protein crystallization

Initial crystals hits were obtained by using the HTXlab platform at EMBL, Grenoble. Initial conditions were optimized by the hanging drop methode at 293 K using EasyXtalTool X-Seal plates (Qiagen). PfuGRHPR was found to crystallize with a mother liquor containing 100 mM sodium acetate, pH 5.2, 15% polyethylene glycol 400 and 100 mM NaCl. PyaGRHPR was found to crystallize with mother liquor containing 1.7 Malonate (Hampton), pH 7.0. For crystallization, 1 ml of mother liquor was placed in the well of the crystallization plate and the drop was formed by mixing 1.5 μl of protein solution at 10 mg.ml^−1^ and 1.5 μl of mother liquor.

Prior to data collection, crystals were cryo-cooled in liquid nitrogen using mother liquor containing 30% PEG 400 as cryo-protectant for PfuGRHPR and 2.5 M Malonate and 15% glycerol for PyaGRHPR. PfuGRHPR derivative crystals were obtained by a 10 seconds soaking of a native crystal in a 2.0 μL solution equivalent to the mother liquor containing 100 mM of GdHPDO3A lanthanide complex.

### Data collection and data processing

X-ray diffraction data were collected on the FIP-BM30A beamline at the European Synchrotron Radiation Facility (ESRF, Grenoble, France) on a single crystal of PyaGRHPR and on the PROXIMA 1 beamline at the French national Synchrotron facility (SOLEIL, Paris, France) on a single crystal of PfuGRHPR for the native data set at 0.9796 Å at 100 K. Gd-derivative data were collected on the same beamline at 1.711 Å.

Diffraction frames were integrated using the program XDS^22^ and the integrated intensities were scaled and merged using the CCP4 programs^23^ SCALA and TRUNCATE, respectively. A summary of the processing statistics is given in Table 2.

PfuGRHPR crystals belong to the I4_1_ space group with one monomer per asymmetric unit. PyaGRHPR crystals belong to the P6_2_22 space group with one dimer per asymmetric unit. Both crystal forms led to a solvent proportion of approximately 75%.

### Experimental SIRAS phasing of PfuGRHPR data

PfuGRHPR structure was solved *de novo* by the SIRAS (Single Isomorphous Replacement with Anomalous Scattering) method. As shown in Table 2, the high value of R_ano_ clearly indicated the presence of GdHPDO3A complex binding sites, which was then confirmed by inspection of the anomalous Patterson map. Gadolinium positions were determined within the asymmetric unit using the program SHELXD^24^. Heavy-atom refinement and initial phasing were performed using the program SHARP^25^. Phases from SHARP were improved by density modification using the CCP4 program SOLOMON^26^ leading to figures of merit of 0.907 after SHARP and density modification, respectively. Automatic model building was performed with the program BUCCANEER^27^.

### Molecular replacement for PyaGRHPR

The structure of PyaGRHPR was determinate by molecular replacement using the 3D structure of the monomer of PhoGRHPR (PDB ID 2DBR) that has a sequence identity of 84% with PyaGRHPR. The calculations were performed with PHASER^28^ using all available diffraction data. The molecular replacement solution has a Z-value of 116 a log-likelihood gain of 672. Automatic model building was performed with the program BUCCANEER^27^.

### Refinement

The models were manually completed and improved in COOT^15^ prior to refinement with PHENIX^29^ using energy minimization and annealing in torsion-angle space in the first round. These models were then optimized through iterative rounds of refinement and model building. At the end stages of the refinement, TLS was used with TLS-groups determined with the TLSMD server^30^,^31^ and hydrogens were added (except for ligands and solvent molecules). The analysis of these final models (Table 2) showed no residues in disallowed regions of the Ramachandran plot (99.7% and 99.3% in preferred and allowed regions for PyaGRHPR and PfuGRHPR respectively).

The nature of the molecules present within the active site was determined by refining the structure in presence of either substrate or product and by inspecting the presence/absence of residuals in mFo-DFc electron density maps. Simulated-annealing sigmaA-weighted Fo - Fc OMIT map were also considered. Associated to the high resolution of the structure of PfuGRHPR, this approach allows us to determine unambiguously that the active site contains glyoxylate. However, in the case of the structure of PyaGRHPR, it was not possible to discriminate between substrate and product.

All figures were prepared using PyMOL (The PyMOL Molecular Graphics System, Version 1.5.0.4 Schrödinger, LLC). Structures superposition were done with THESEUS^32^. Crystallographic software support is provided by SBGrid^33^.

The atomic coordinates and measured structure factor amplitudes for PfuGRHPR and PyaGRHPR have been deposited in the Protein Data Bank with accession code 5AOV and 5AOW, respectively.

## Additional Information

**How to cite this article**: Lassalle, L. *et al*. New insights into the mechanism of substrates trafficking in Glyoxylate/Hydroxypyruvate reductases. *Sci. Rep.* **6**, 20629; doi: 10.1038/srep20629 (2016).

## Supplementary Material

Supplementary Information

## Figures and Tables

**Table 1:** Kinetic parameters of PfuGRHPR, PhoGRHPR and PyaGRHPR.

Hydroxypyruvate	PfuGRHPR		PhoGRHPR		PyaGRHPR	
	NADPH	NADH	NADPH	NADH	NADPH	NADH
k_cat_/K_M_ (s^−1^/mM)	15 +/− 5	75 +/− 50	30 +/− 15	65 +/− 20	40 +/− 20	60 +/− 25
K_M_ (μM)	90 +/− 25	140 +/− 55	20 +/− 10	70 +/− 11	165 +/− 55	410 +/− 110
k_cat_ (s^−1^)	1.20 +/− 0.05	10 +/− 5	0.60 +/− 0.05	4.5 +/− 0.5	6.5 +/− 1.0	25 +/− 5
K_I_ (μM)	none	350 +/− 140	none	3820 +/− 680	2000 +/− 740	760 +/− 200
**Glyoxylate**						
k_cat_/K_M_ (s^−1^/mM)	5.5 +/− 1.0	7.0 +/− 0.5	1.0 +/− 0.5	25 +/− 5	3.5 +/− 1.0	3.0 +/− 0.5
K_M_ (μM)	220 +/− 30	1800 +/− 25	480 +/− 80	160 +/− 25	315 +/− 50	1500 +/− 140
k_cat_ (s^−1^)	1.20 +/− 0.05	13.0 +/− 0.5	0.60 +/− 0.02	4.0 +/− 0.5	1.1 +/− 0.04	4.8 +/− 0.2
K_I_ (μM)	none	none	none	none	none	none

Assays were performed as described under “Methods”. The indicated errors are standard errors.

**Table 2:** Data collection and refinement statistics.

	PfuGRHPR	PfuGRHPR SAD	PyaGRHPR.
Data collection			
Space group	I4_1_	I4_1_	P6_2_22
a,b,c (Å)	114.58 – 114.58 – 118.12	115.56 – 155.56 – 118.99	141.06 –141.06 –260.79
Resolution range (Å)	47.73 – 1.40 (1.48 –1.40)	48.10 – 2.10 (2.21– 2.10)	47.97 – 2.00 (2.11– 2.00)
R_sym_ (%)	4.6 (108.0)	5.3 (14.6)	17.0 (188.0)
R_pim_ (%)^(a)^	2.3 (57.5)	5.5 (14.6)	6.2 (68.3)
CC_1/2_ (%)^(b)^	100.0 (54.0)	100.0 (93.0)	100.0 (52.7)
I/σ (I)	14.3 (1.7)	12.6 (4.2)	11.7 (1.4)
Multiplicity	3.7 (3.2)	4.9 (3.3)	7.3 (7.3)
Completeness (%)	99.4 (96.6)	99.2 (95.8)	99.8 (99.3)
Refinement			
Resolution (Å)	1.40		2.00
No. reflections	148 562		103 194
R_factor_ (%)/ R_free_ (%)	13.2/14.3		15.1/17.9
No. atoms			
Protein	2956		5334
Water	432		670
Ligands	150		269
*B*-factors			
Protein	27.3		40.8
Water	45.5		53.5
Ligands	31.9		42.5
R.m.s. deviations			
Bond lengths (Å)	0.022		0.006
Bond angles (°)	1.905		0.899

(a) 
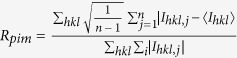
 with *I*_*hkl,j*_ is the *j^th^* intensity measurement of reflection *hkl* and 〈*I*〉 is the average intensity from multiple observations. n represents the multiplicity of the measurements^34^. (b) CC½ = Correlation coefficient between random half datasets^35^,^36^,^37^,^38^

**Table 3:** The table summarized the interactions between catalytic residues and D-glycerate or glyoxylate in different GRHPRs.

Residue		D-Glycerate			Glyoxylate	
Number	Atom	Atom	Distances in PyaGRHPR structure	Distances in hGRHPR structure	Atom	Distances in PfuGRHPR structure
His 288	NE2	O3	2.83 (2.89)	2.80 (2.54)	O1	2.73
Arg 241	NH1	O3	2.66 (2.62)	2.77 (3.05)	O1	2.52
Arg 241	NH2	O2	2.80 (2.61)	3.00 (2.82)	O2	2.81
Gly 77	N	O2	3.07 (3.12)	2.70 (2.86)	O2	2.70
Val 76	N	O1	2.96 (3.02)	2.90 (2.75)	O3	2.79
Ser 291	OG	O4	3.99 (3.81)	2.92 (2.67)		

Distances between catalytic residues and substrate molecules are indicated in angströms. For PyaGRHPR and hGRHPR, the indicated values refer to the individual measures of the distances and correspond to the different occupied active sites present in the asymmetric unit.
