# Erratum: New insights into the mechanism of substrates trafficking in Glyoxylate/Hydroxypyruvate reductases

**DOI:** 10.1038/srep23879

**Published:** 2016-04-20

**Authors:** Louise Lassalle, Sylvain Engilberge, Dominique Madern, Pierre Vauclare, Bruno Franzetti, Eric Girard

Scientific Reports 6: Article number: 2062910.1038/srep20629; published online: 02112016; updated: 04202016.

The original version of this Article contained errors in the Received ‘7 October 2015’ and Accepted dates ’06 January 2016’ which were incorrectly given as ‘10 July 2015’ and ‘01 June 2016’ respectively. These errors have now been corrected in the PDF and HTML versions of the Article.

