# Supplementary material for: New insights into the mechanism of substrates trafficking in Glyoxylate/Hydroxypyruvate reductases
Source: Sci Rep. 2016 Feb 11;6:20629. doi: 10.1038/srep20629 (PMC4749974; doi:10.1038/srep20629)

New insights into the mechanism of substrates trafficking in Glyoxylate/Hydroxypyruvate reductases.

Louise Lassalle[123], Sylvain Engilberge[123], Dominique Madern[213], Pierre Vauclare[213], Bruno Franzetti[213], Eric Girard[312]

1 Univ. Grenoble Alpes, IBS, F-38044 Grenoble, France

2 CNRS, IBS, F-38044 Grenoble, France

3 CEA, IBS, F-38044 Grenoble, France

ONLINE-ONLY MATERIAL

In this section we have included supporting information concerning one figure.

Supplementary Figure S1:

For all panels, relative specific activity as a function of temperature is represented for P. furiosus GRHPR (circle, green), P. horikoshii GRHPR (square, red) and P. yayanosii GRHPR (triangle, blue) for (A) Hydroxypyruvate reductase (HPR) activity with NADH, (B) Hydroxypyruvate reductase activity with NADPH, (C) Glyoxylate reductase (GR) activity with NADH and (D) Glyoxylate reductase activity with NADPH. Standard deviation bars are not visible when they are smaller than the font size used for the data point. Assays were performed as described under "Methods"**.**

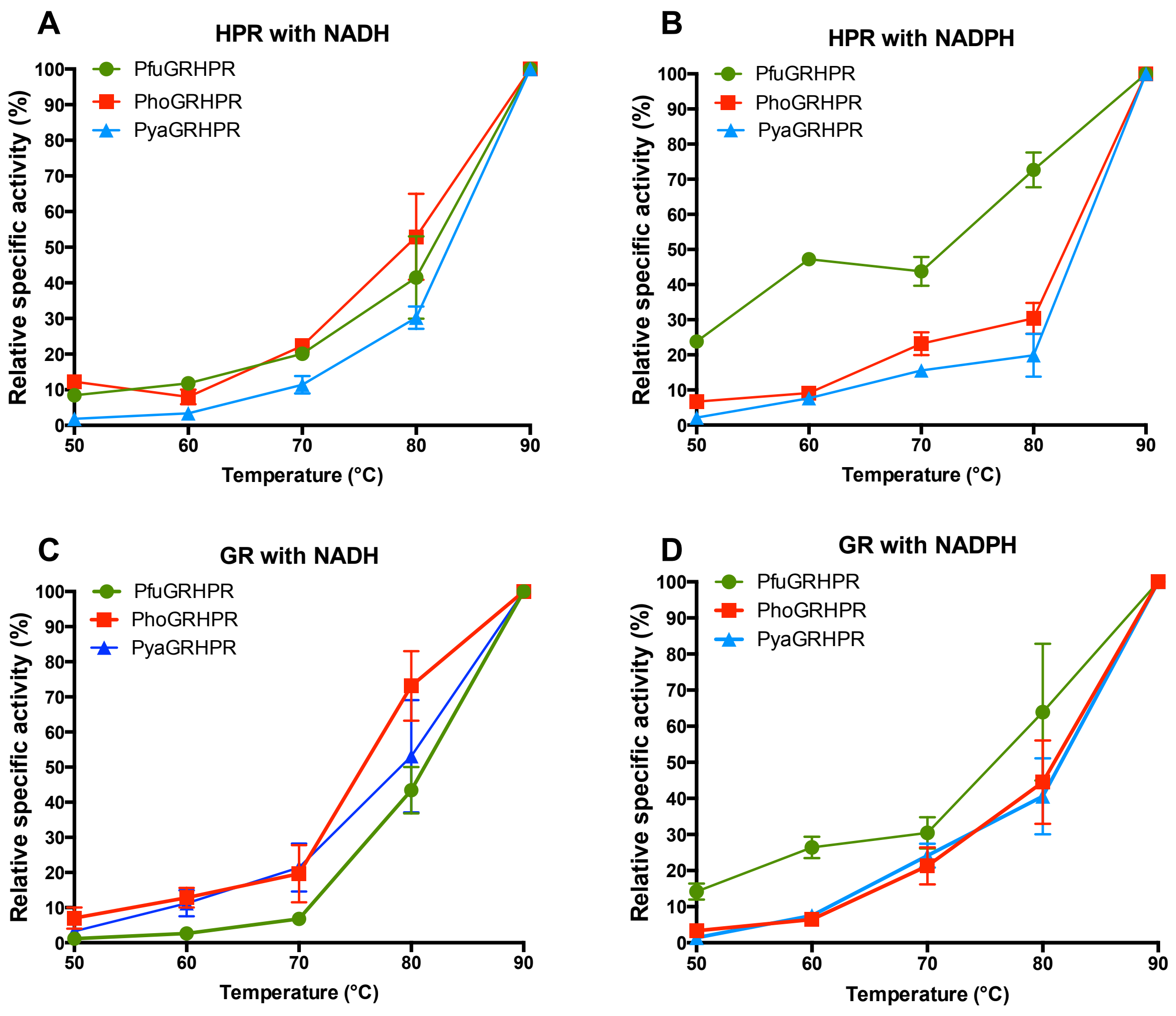

Supplement: Supplementary Information [file srep20629-s1.pdf]
